# Supplementary material for: Multimodal measures of spontaneous brain activity reveal both common and divergent patterns of cortical functional organization
Source: Nat Commun. 2024 Jan 3;15:229. doi: 10.1038/s41467-023-44363-z (PMC10764905; doi:10.1038/s41467-023-44363-z)
Supplement: Supplementary file 1 — Supplementary Information [file 41467_2023_44363_MOESM1_ESM.pdf]

Supplementary Information for:

Multimodal measures of spontaneous brain activity reveal both common and divergent patterns of cortical functional organization

Hadi Vafaii[1,✉], Francesca Mandino[2], Gabriel Desrosiers-Grégoire[3,4], David O'Connor[5], Marija Markicevic[2], Xilin Shen[2], Xinxin Ge[6], Peter Herman[2], Fahmeed Hyder[2], Xenophon Papademetris[2,5,7], Mallar Chakravarty[3,4,8,9], Michael C. Crair[10–12], R. Todd Constable[2,5,13], Evelyn MR. Lake[2,5,✉,*], and Luiz Pessoa[14–16,✉,*]

[1]Department of Physics, University of Maryland, College Park, MD, 20742, USA
[2]Department of Radiology and Biomedical Imaging, Yale School of Medicine, New Haven, CT, 06520, USA
[3]Comp. Brain Anatomy Laboratory, Cerebral Imaging Center, Douglas Mental Health Univ. Institute, Montreal, QC, H4H 1R3, Canada
[4]Integrated Program in Neuroscience, McGill University, Montreal, QC, H3A 0G4, Canada
[5]Department of Biomedical Engineering, Yale University, New Haven, CT, 06520, USA
[6]Department of Physiology, School of Medicine, University of California San Francisco, San Francisco, CA, 94143, USA
[7]Section of Biomedical Informatics & Data Science, Yale School of Medicine, New Haven, CT, 06520, USA
[8]Department of Psychiatry, McGill University, Montreal, QC, H3A 0G4, Canada
[9]Department of Biological and Biomedical Engineering, McGill University, Montreal, QC, H3A 0G4, Canada
[10]Department of Neuroscience, Yale School of Medicine, New Haven, CT, 06510, USA
[11]Kavli Institute for Neuroscience, Yale School of Medicine, New Haven, CT, 06510, USA
[12]Department of Ophthalmology and Visual Science, Yale School of Medicine, New Haven, CT, 06510, USA
[13]Department of Neurosurgery, Yale School of Medicine, New Haven, CT, 06510, USA
[14]Department of Psychology, University of Maryland, College Park, MD, 20742, USA
[15]Department of Electrical and Computer Engineering, University of Maryland, College Park, MD, 20742, USA
[16]Maryland Neuroimaging Center, University of Maryland, College Park, MD, 20742, USA

✉*Correspondence to:* vafaii@umd.edu, evelyn.lake@yale.edu, pessoa@umd.edu
*These authors jointly supervised this work: Evelyn MR. Lake, Luiz Pessoa

Supplementary Discussion

Choosing number of communities

Clustering is in the eyes of the beholder (or the algorithm) [1]. Community detection is inherently ill-defined: algorithms do not find communities, what they do is *"define"* communities and later find them according to their definition. Here, we employed a mixed-membership stochastic blockmodel [2], where each community corresponds to a *latent functional role* (see [3] for an extensive review). Choosing a specific number of communities is thus equivalent to deciding how many functional roles (or clusters) best describe the observed graphs. In this sense, there is no *"true"* number of communities. Therefore, instead of focusing on a single result as a true decomposition, we explored network organization at different levels of granularity. We decided the number of communities empirically, using criteria such as bilateral symmetry. At the most coarse level, our $K = 3$ communities recapitulated previous seed-based reports in Ca^{2+} data and coarse fMRI-ICA findings. Our $K = 7$ decomposition was similar to previous fMRI-ICA reports [4, 5]. Overall, the highly bilateral nature of the resulting community structure (observed for up to $K = 20$ for Ca^{2+}) and their similarity to previous reports increased our confidence about our results.

Thresholding the graphs

The algorithm used in this study requires binary graphs as its input [6], which necessitates choosing an edge-filtering threshold. To mitigate this, we employed an approach known as proportional thresholding which is known to perform better than alternatives such as absolute thresholding [7]. In addition, theoretical work has shown that network topology is highly robust against different thresholds [8]. Here we reported results at a graph density of $d = 15\%$. To ensure the robustness of our findings to this arbitrary choice, we also explored densities going from $d = 10\%$ all the way up to $d = 25\%$ with incremental steps of 5%. Empirically, we found that our results were robust to the choice of edge density, as well as other hyperparameters. Overall, our data partially confirmed previous theoretical and simulation work that network topology is robust across a wide range of sparsity levels.

Region of interest (ROI) definition scheme

We started by using brain region masks from Allen Reference Atlas (ARA [9]) as our initial choice of ROIs but observed some mismatch between functional and ARA parcellations. This is probably because ARA regions were delineated using various anatomical and structural criteria; but crucially, function was not one of them. Here, we introduced a new parcellation scheme illustrated in Fig. 1c and d, which increased the robustness of our results. In conclusion, we found that spatially homogeneous ROIs worked well for the purpose of functional network construction, consistent with previous reports in humans [10].

Defining appropriate ROIs for our multimodal dataset was challenging because different modalities occupy spaces with different geometries. Namely, fMRI data is defined within a 3D volumetric space, while mesoscopic Ca^{2+} imaging data exists only on the 2D cortical surface. To address this, we started from the 2D space of cortical flatmap (Fig. 1c; step I), which fits Ca^{2+} data well. We then added depth to obtain 3D volumetric ROIs (Fig. 1c; step II), suitable for fMRI data. In the depth dimension, we included cortical layers 1 to 4. Crucially, our goal was not to obtain layer-specific results (BOLD resolution was 0.4 *mm* isotropic). Instead, we wanted to consider depths of the cortex that most likely contribute to Ca^{2+} signal [11–15], which would render our network-level cross-modality comparisons more meaningful.

Comparing entropy to other measures of node diversity

Brain regions of high functional diversity are more likely to participate in multiple networks. This region-level property can be characterized using appropriate node centrality measures. Here, we took advantage of having access to continuous membership values and defined node entropy centrality (Fig. 5). Another measure, *"participation coefficient"* [16–20], has also been commonly used to quantify a similar concept: a node's participation coefficient measures how well-distributed its links are among different communities. Large participation coefficients indicate higher amounts of link diversity, which could be potentially related to high membership diversity. To understand the relationship between the two measures, we visualized their spatial patterns and found that entropy and participation coefficient maps were largely in agreement (Fig. 8). The node-wise correlation between entropy and participation coefficient was $r = 0.70 \pm 0.09$, BOLD; $r = 0.77 \pm 0.12$, Ca^{2+}_{slow} ; $r = 0.47 \pm 0.28$, Ca^{2+}_{fast} .

It is worth noting that node entropy and participation coefficient are defined in very different ways. Entropy is computed from membership probability vectors within our overlapping framework; whereas, participation coefficient depends on how links are distributed across communities within a disjoint framework. Despite this, the two measures were highly correlated indicating that they probably capture similar underlying phenomena.

Mouse cortical areas visible from the top view

The goal of the present study was to examine the cross-modality correspondences between mouse cortical networks derived from our simultaneous fMRI-BOLD and wide-field Ca^{2+} imaging dataset (Fig. 1a). As such, we restricted our analyses to the cortical surface that appears in the Ca^{2+} imaging field-of-view (Fig. 1d). We provided both a coarse (Fig. 2b), and a fine (Supplementary Fig. 1b) delineation of the mouse cortical areas visible in this top view. In these delineations, solid lines correspond to anatomical regions as defined in the CCFv3 Allen reference atlas [9]. In addition, dashed lines approximately correspond to functionally defined subregions in the secondary motor area [21, 22]. The abbreviations for all the brain regions that appear in Fig. 2b and Fig. 1b are provided in Table 1 below. Please refer to the CCFv3 publication for the full list of brain regions in Allen reference atlas ontology [9].

Supplementary Table & Figures

In this section, we include a Supplementary table that contains information about brain region abbreviations, along with Supplementary figures that provide additional support for the results in the main paper.

Supplementary Table 1: Brain region abbreviations.

Abbrev.	Full structure name
ACA	Anterior cingulate area
AUD	Auditory areas
AUDd	Dorsal auditory area
AUDp	Primary auditory area
AUDpo	Posterior auditory area
AUDv	Ventral auditory area
MO	Somatomotor areas
MOs	Secondary motor area
MOp	Primary motor area
PL	Prelimbic area
PTLp	Posterior parietal association areas
RSP	Retrosplenial area
RSPagl	Retrosplenial area, lateral agranular part
RSPd	Retrosplenial area, dorsal part
RSPv	Retrosplenial area, ventral part
SS	Somatosensory areas
SSp	Primary somatosensory area
SSp-n	Primary somatosensory area, nose
SSp-bfd	Primary somatosensory area, barrel field
SSp-ll	Primary somatosensory area, lower limb
SSp-m	Primary somatosensory area, mouth
SSp-ul	Primary somatosensory area, upper limb
SSp-tr	Primary somatosensory area, trunk
SSp-un	Primary somatosensory area, unassigned
SSs	Supplemental somatosensory area
TEa	Temporal association areas
VIS	Visual areas
VISal	Anterolateral visual area
VISam	Anteromedial visual area
VISl	Lateral visual area
VISp	Primary visual area
VISpl	Posterolateral visual area
VISpm	posteromedial visual area
VISli	Laterointermediate area
VISpor	Postrhinal area

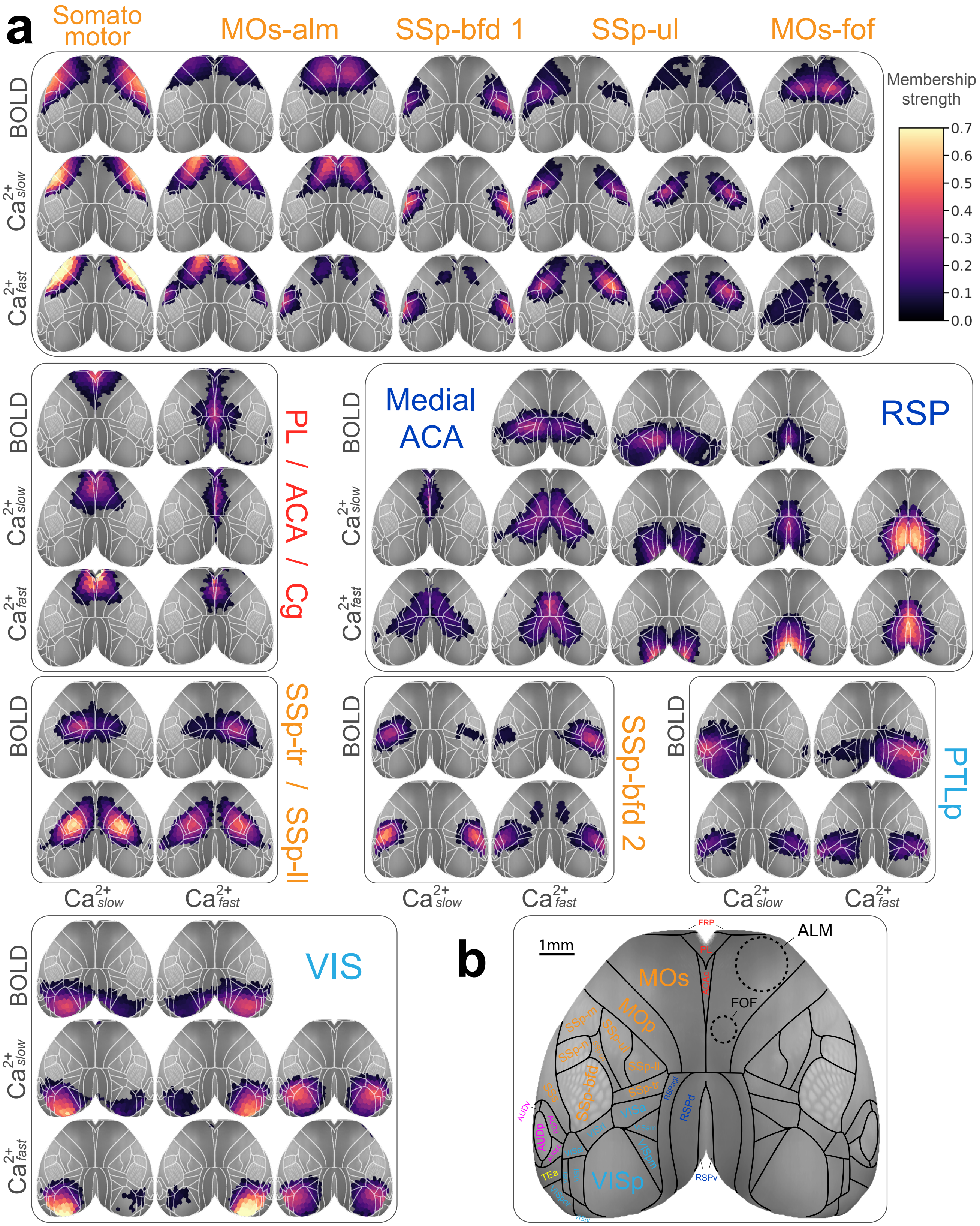

Supplementary Figure 1: $K = 20$ network decomposition. **(a)** Even at $K = 20$, most networks maintain their bilateral symmetry, especially for Ca^{2+} . A network centered around FOF appears as its own separate network for BOLD, similar to the $K = 7$ solution (top-right). In contrast, this network did not appear separately for Ca^{2+} , even at $K = 20$. Instead, FOF partially overlaps with a large medial Ca^{2+} network that spans parts of SSp-tr/ll. **(b)** Fine divisions of the cortical regions in Allen reference atlas, along with anterior lateral motor area, ALM; and frontal orienting field, FOF (dashed lines). Label colors inspired from Figure 1F in Harris et al. [23]. A complete list of brain region abbreviations are provided in Table 1. Compare with Fig. 2 for $K = 3$ and $K = 7$ solutions. Source data are provided as a Source Data file.

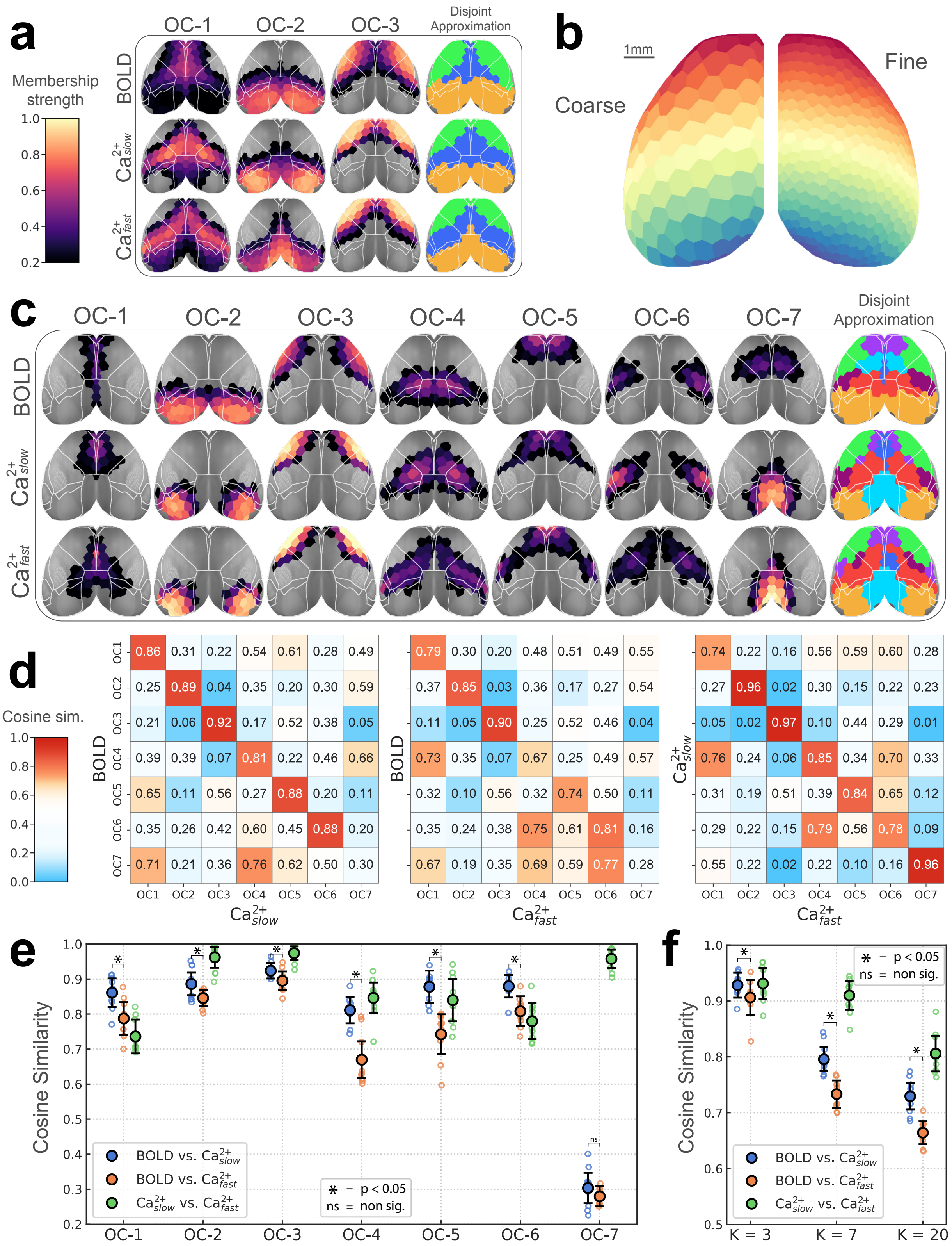

Supplementary Figure 2: Network structure is robust to the choice of ROI granularity. **(a, c-f)** Similar to Fig. 2. **(b)** Left, results with coarse ROIs are presented here; Right, fine ROIs were used for the main results. **(e-f)** Empty circles correspond to individual animals ($N = 10$); large solid circles are the group average. Error bars are 95% confidence intervals based on hierarchical bootstrap (Methods). Comparison of BOLD and Ca^{2+}_{slow} networks (paired permutation test, two-sided, $p < 0.05$, Holm-Bonferroni corrected). The exact p-values were as follows (uncorrected): OC-1, 4.0×10^{-3}, OC-2, 1.1×10^{-3}, OC-3, 3.2×10^{-3}, OC-4 and 5, 2.0×10^{-6}, OC-6, 1.1×10^{-3}, OC-7, 8.7×10^{-2}; and, $K = 3$, 1.0×10^{-2}, $K = 7$ and 20, 2.0×10^{-6}. OC, overlapping community. See also Supplementary Fig. 3. Source data are provided as a Source Data file.

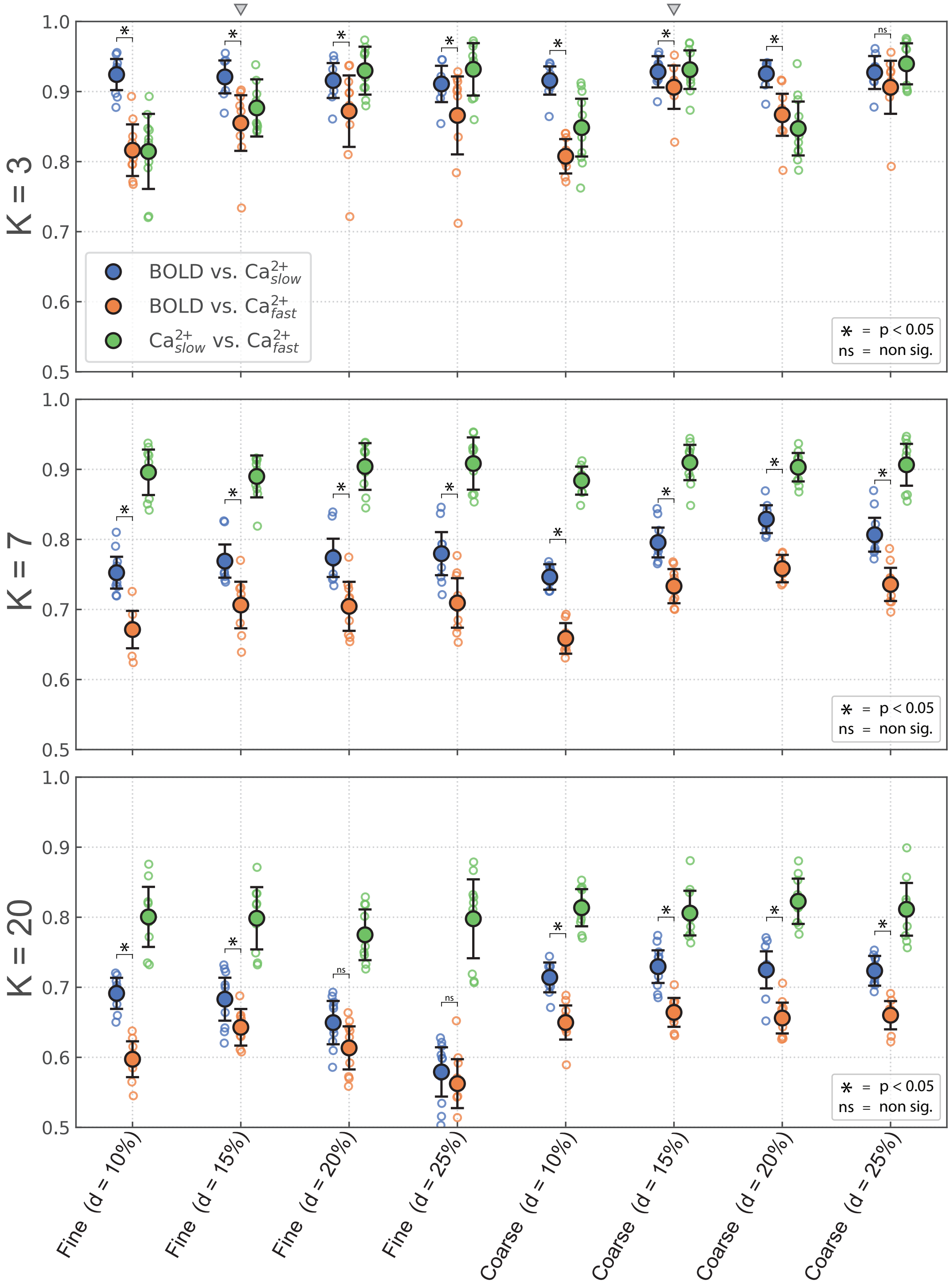

Supplementary Figure 3: BOLD network organization is more similar to Ca^{2+}_{slow} than it is to Ca^{2+}_{fast} . This is a robust finding in the present study, as it is reproduced over a large combinatorial space of analysis conditions. The y-axis is cosine similarity and the x-axis corresponds to different conditions. See Supplementary Fig. 2b for a visual comparison of coarse versus fine ROIs. d is graph density after edge-filtering is applied. Small triangles indicate our choices for the main results: $d = 15\%$, fine ROIs. Empty circles correspond to individual animals ($N = 10$); large solid circles are the group average. To compare conditions, we conducted paired permutation tests (two-sided, $p < 0.05$, Holm-Bonferroni corrected). Error bars are 95% confidence intervals based on hierarchical bootstrap (Methods). See also Fig. 2 and Supplementary Fig. 2. Source data are provided as a Source Data file.

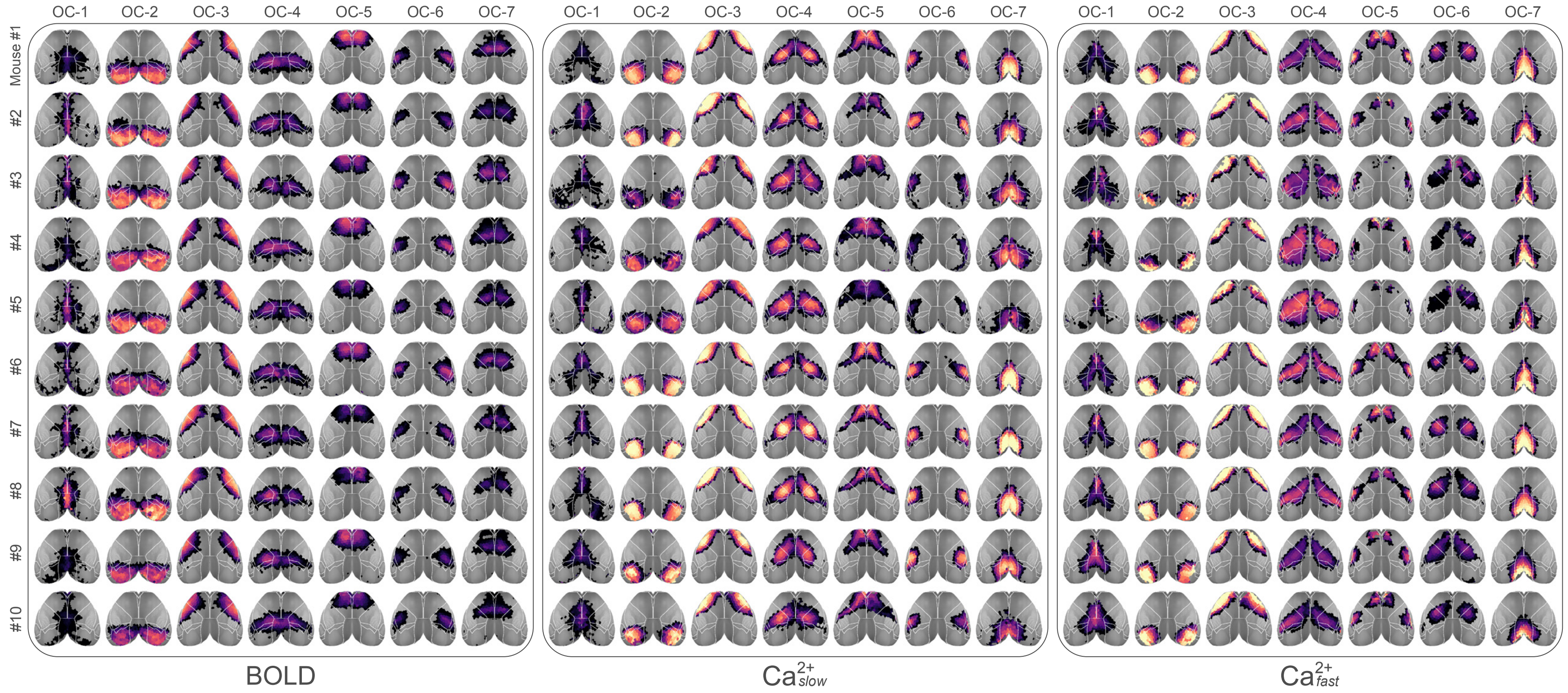

Supplementary Figure 4: Overlapping networks at the level of individuals. Each mouse was highly sampled (Fig. 1b), which allowed robust estimation of individualized networks. The high similarity of network structures across individuals is anticipated; however, there are still visible differences. Overall, the reproducibility of individualized networks is notable and lends additional support to our group results (Fig. 2c). Source data are provided as a Source Data file.

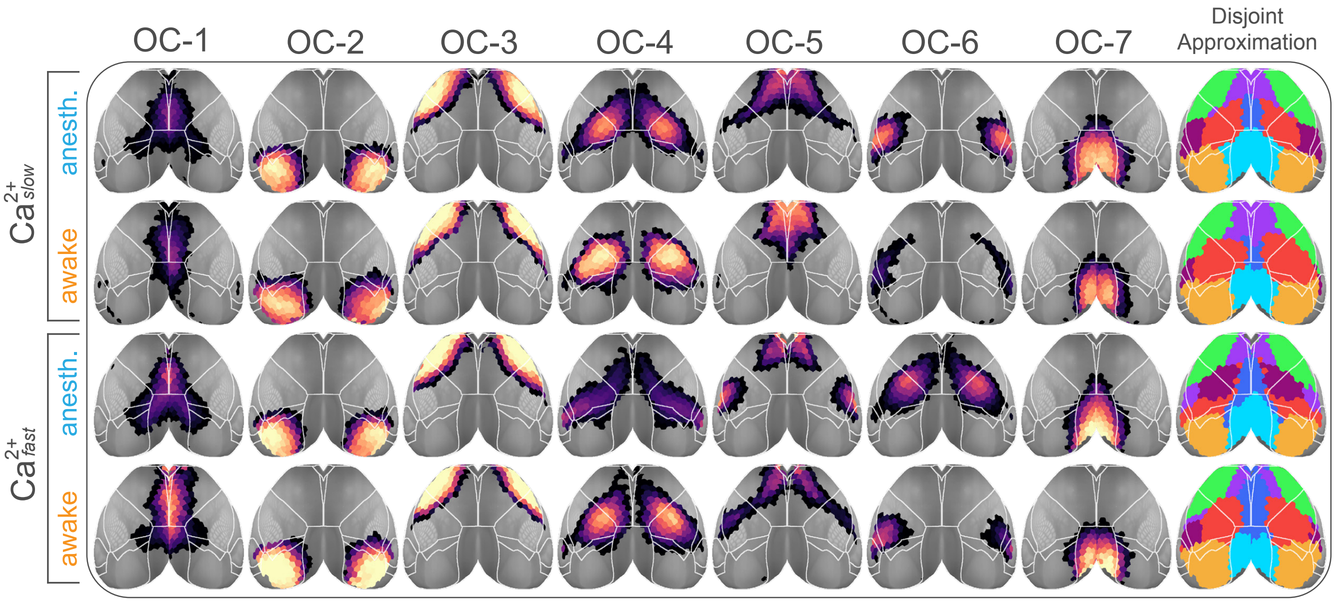

Supplementary Figure 5: Awake results (Ca^{2+} only). We report exploratory analysis in a group of $N = 5$ animals for which we had Ca^{2+} recordings in both anesthetized and awake states. Along with the awake results, we also plotted group results obtained from the same subset of $N = 5$ animals in the anesthetized state. The overall community structure is preserved irrespective of animal state, which is expected due to the low dosage of anesthetics used in our study (Methods). Despite the overall similarities, we still observe some differences, most visibly in Ca^{2+}_{fast} . Further, the group result obtained from half of the anesthetized dataset is almost identical to the full $N = 10$ results shown in Fig. 2c, indicating the robustness and reproducibility of our approach. Source data are provided as a Source Data file.

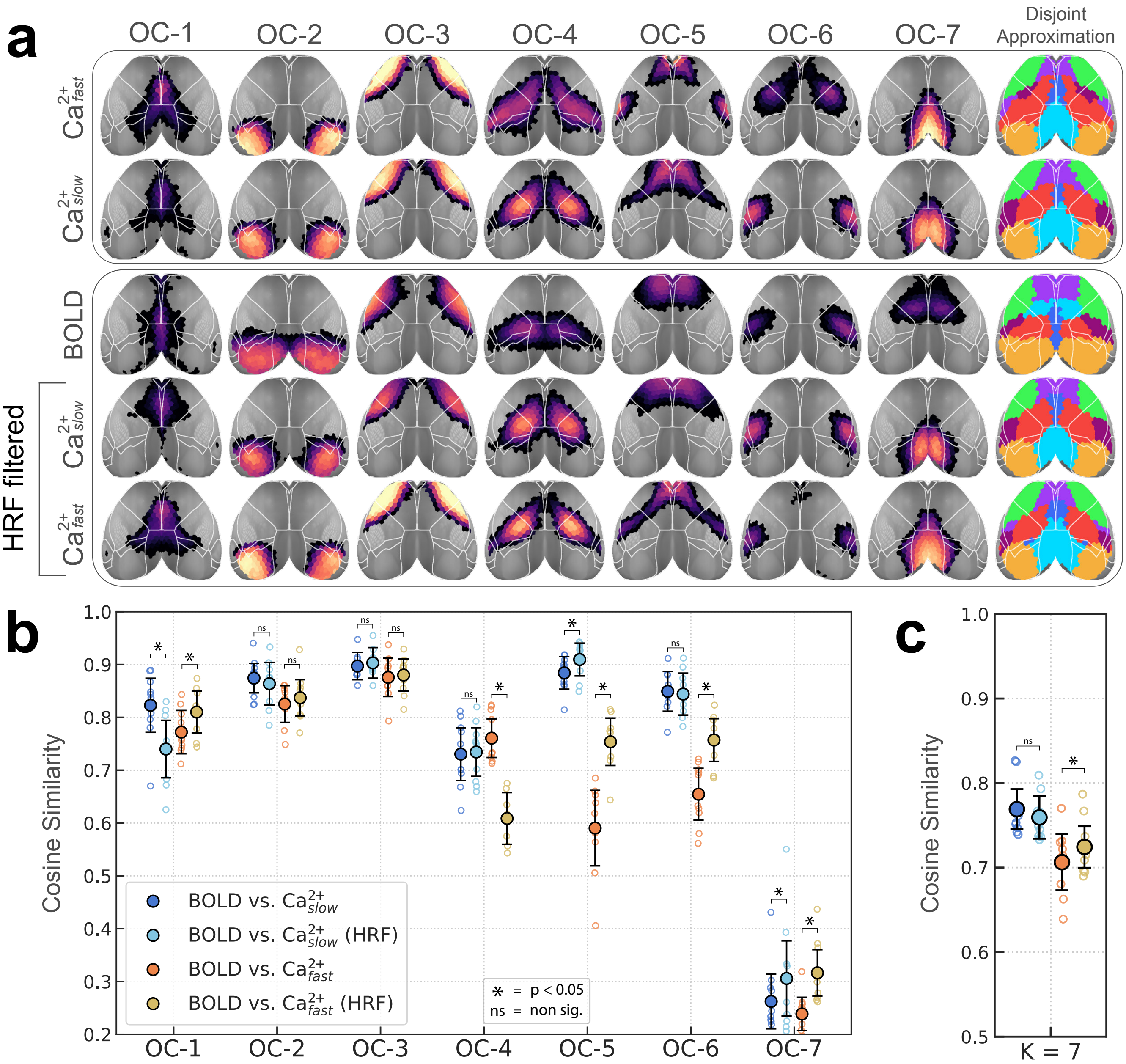

Supplementary Figure 6: Filtering Ca^{2+} data with a hemodynamic response function (HRF) results in modest changes in network structure. We applied the gamma-variate model of Ma et al. [24] using parameters previously published by us [25]. We then inferred the community structure of HRF-filtered Ca^{2+} data using steps otherwise identical to those in the main results (Methods). **(a)** The top three rows are reproduced from Fig. 2c to facilitate visual comparison, with a reordering of rows that puts BOLD adjacent to HRF-Ca^{2+} results in the bottom two rows. **(b, c)** Comparison of BOLD vs. HRF-Ca^{2+} results relative to BOLD vs. Ca^{2+} results from the main paper. **(b)** Per-network; and, **(c)** Overall cosine similarity. **(b-c)** Empty circles correspond to individual animals ($N = 10$); large solid circles are the group average. Error bars are 95% confidence intervals based on hierarchical bootstrap (Methods). To compare conditions, we conducted paired permutation tests (two-sided, $p < 0.05$, Holm-Bonferroni corrected). Source data are provided, which include exact p-values (uncorrected).

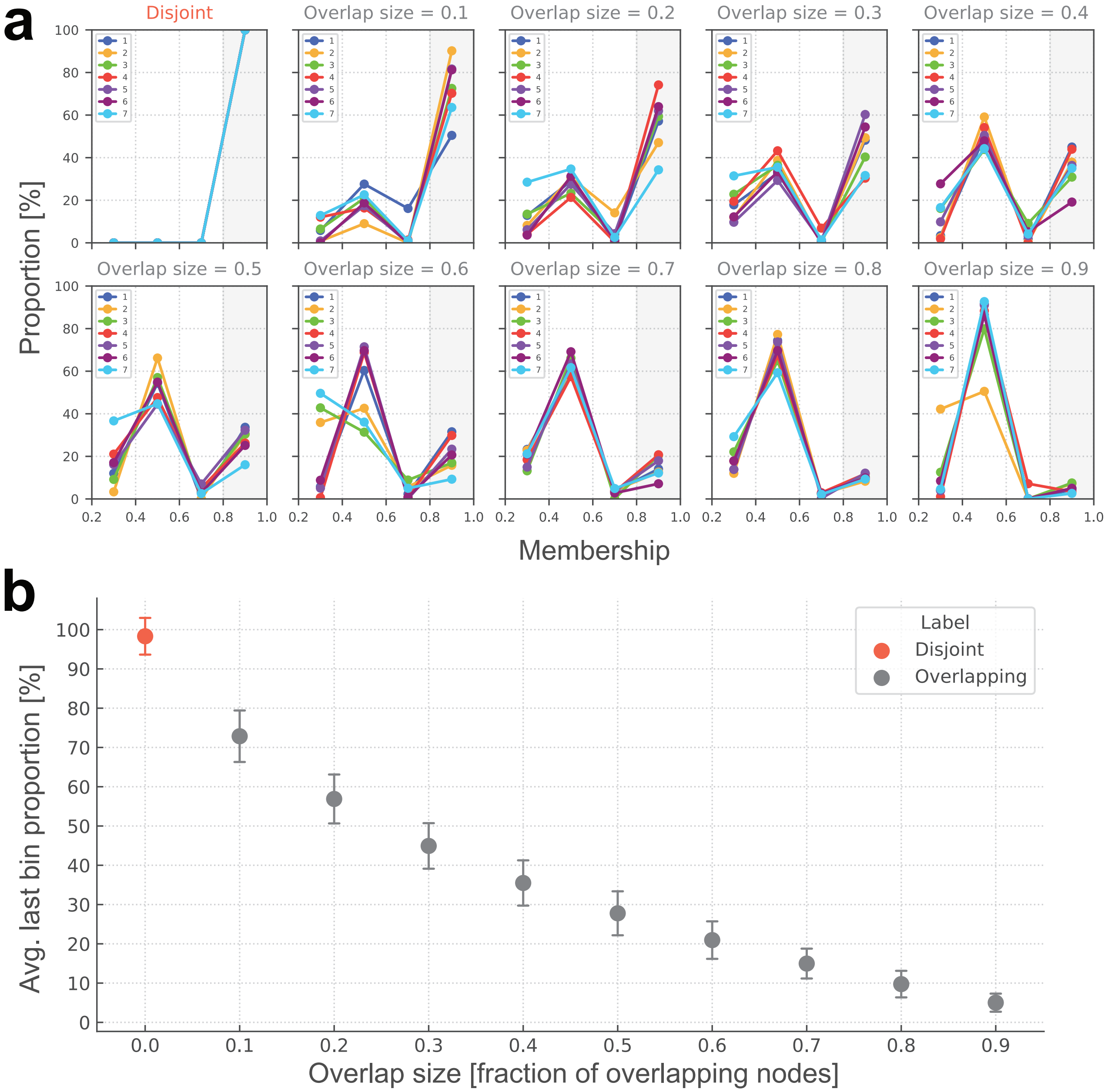

Supplementary Figure 7: Verifying our analysis procedure using synthetic LFR graphs [26]. **(a)** To compute the distribution of node memberships, we divided the interval $(0.2, 1.0]$ into four bins of equal width. We chose this binning scheme because it allowed us to distinguish between disjoint and overlapping graphs: for disjoint graphs, the bin corresponding to strong membership values (i.e., last bin) had a proportion of 100%, and the proportions were 0% elsewhere. Overlap size, or fraction of overlapping nodes, is a tunable parameter in LFR graphs. Here we show how membership distributions change as a function of overlap size. **(b)** We averaged the last bin proportions across all communities to get a single statistic per graph. Its mean over thousands of simulated graphs is shown here. The average last bin proportion drops as overlap size increases, thus making it a reliable proxy for network overlap size. Error bars indicate standard deviation. Compare with Fig. 3.

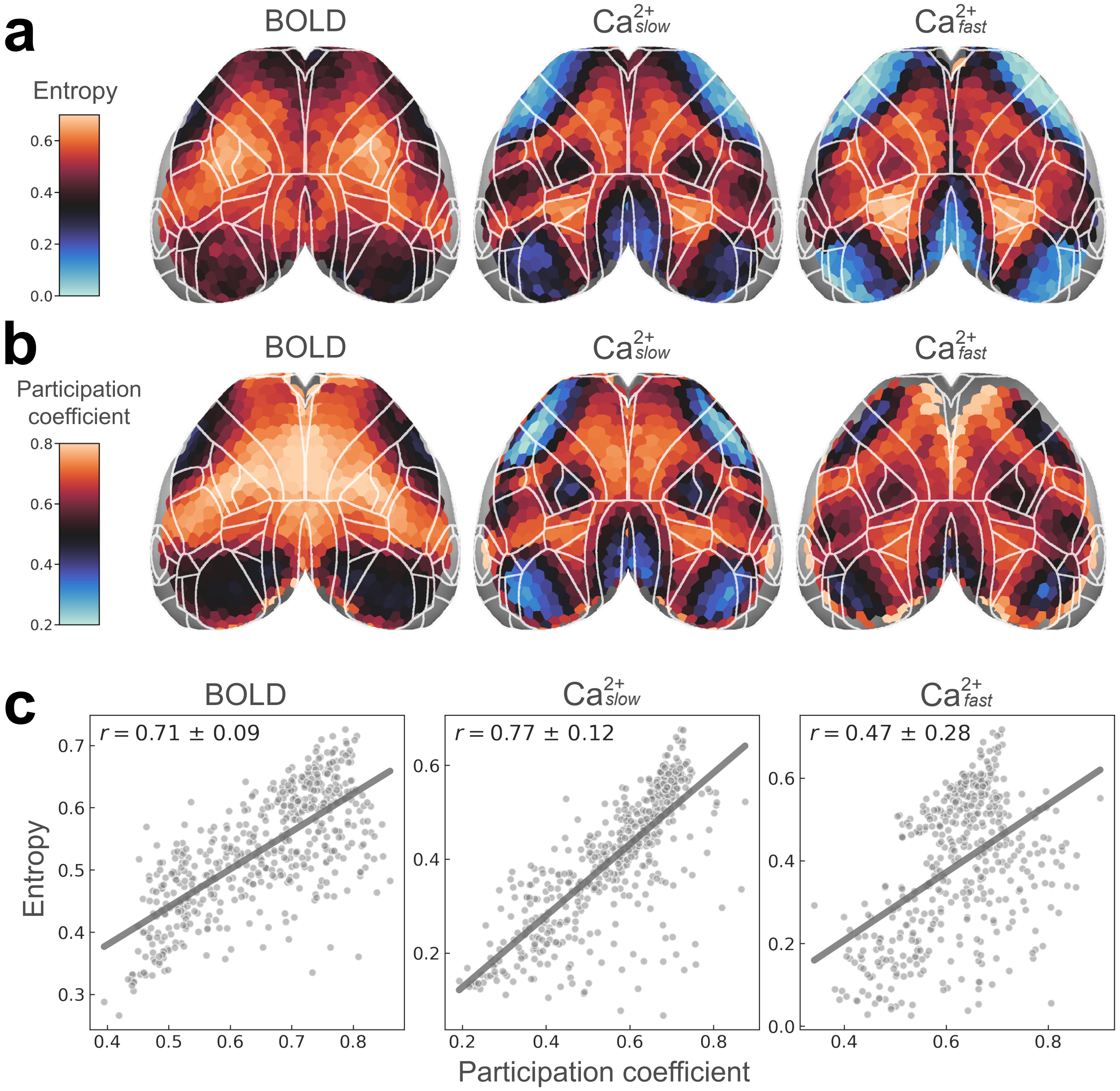

Supplementary Figure 8: Entropy and participation coefficient uncover similar spatial patterns. **(a)** Here, we show entropy maps with actual values (compare with Fig. 5 for the rank-ordered version). Note the positive shift in BOLD values. **(b)** Participation coefficient is commonly used to quantify how a node's links are distributed across (disjoint) communities [16–20]. To compute participation coefficients, we used the disjoint approximation obtained from taking the maximum membership of a given node (see the last column in Fig. 2). Note that for low-degree nodes with degree < 7, participation coefficient estimates become unreliable. For example, see frontal regions in Ca$^{2+}_{fast}$. **(c)** The two centrality measures exhibit a positive (Pearson) correlation (variability obtained based on hierarchical bootstrapping; Methods). The concordance between these measures is most clearly visible for Ca$^{2+}_{slow}$, but also for BOLD to some extent. Source data are provided as a Source Data file.

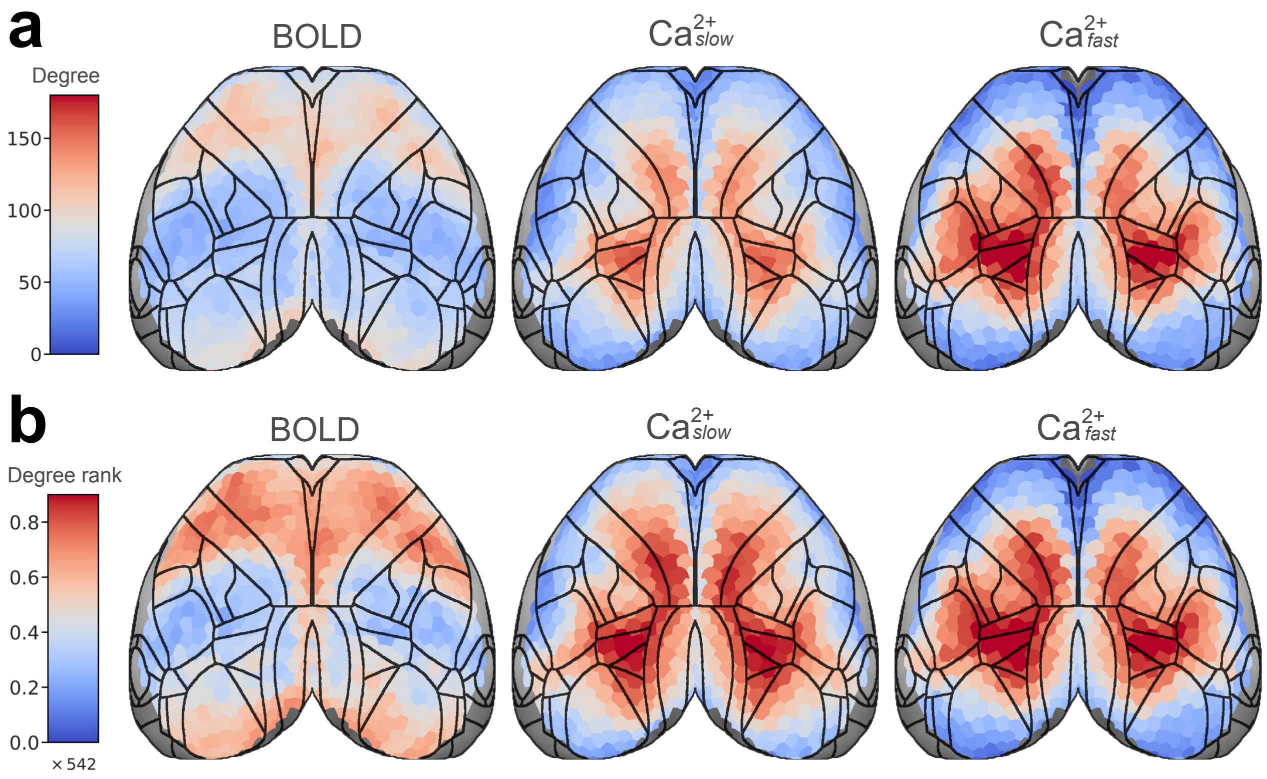

Supplementary Figure 9: **(a)** Both the magnitude and spatial patterns of degree centrality values are different across modalities. **(b)** Degree ranks are reproduced from Fig. 6 to facilitate visual comparison. Source data are provided as a Source Data file.

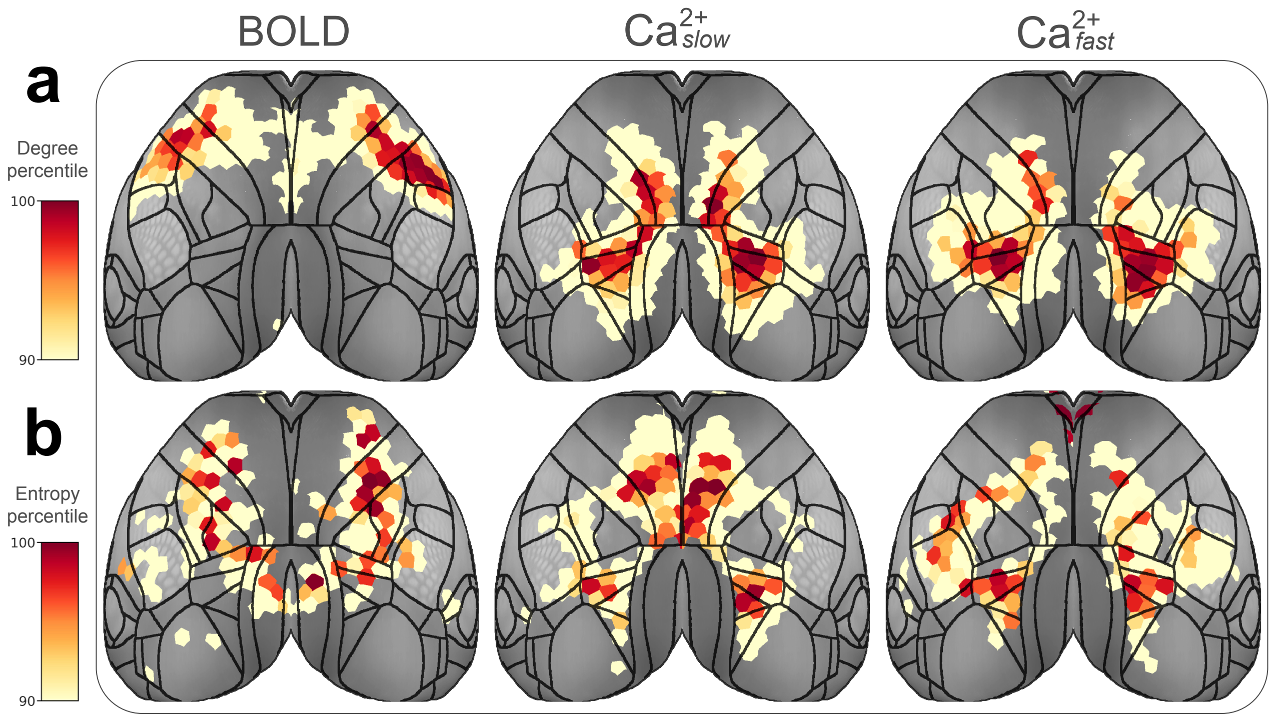

Supplementary Figure 10: Percentile maps are obtained by calculating t-statistics (hierarchical bootstrapping, see Methods) followed by rank-ordering. Values below 80% are not shown. **(a)** Degree **(b)** Entropy. Related to Figs. 5 and 6. Source data are provided as a Source Data file.

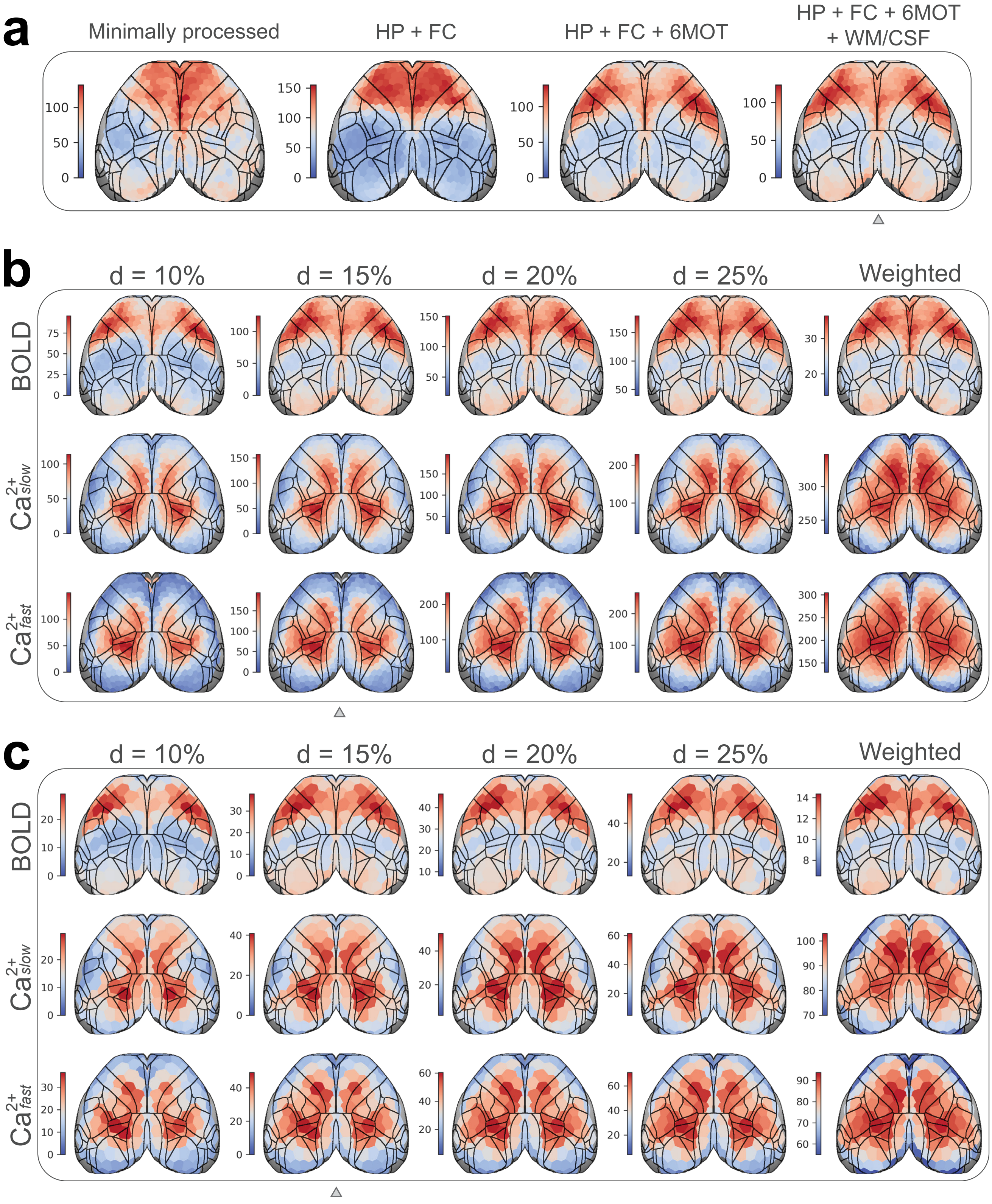

Supplementary Figure 11: Dependence of degree centrality to preprocessing and analysis choices. **(a)** Spatial patterns of average node degree are somewhat altered depending on which BOLD preprocessing steps are used. Minimally processed, motion correction (rigid transformations) and detrending; HP, high-pass filtering (0.01 Hz); FC, frame censoring; 6MOT, motion regression (6 parameters); WM/CSF, average signal from white matter and ventricles regressed out. **(b)** Degree patterns are robust to the choice of edge filtering threshold. Difference thresholds result in different scales, but the spatial patterns remain relatively similar. **(c)** Similar to B but for a coarse parcellation (see Supplementary Fig. 2b). Small triangles indicate the pipeline and parameters used for the main results. Related to Fig. 6. Source data are provided as a Source Data file.

Supplementary References

[1] Ulrike Von Luxburg, Robert C Williamson, and Isabelle Guyon. "Clustering: Science or art?" In: *Proceedings of ICML workshop on unsupervised and transfer learning*. JMLR Workshop and Conference Proceedings. 2012, pp. 65–79. URL: http://proceedings.mlr.press/v27/luxburg12a.html.

[2] Edo M Airoldi et al. "Mixed membership stochastic blockmodels". In: *Advances in neural information processing systems* 21 (2008). URL: https://proceedings.neurips.cc/paper/2008/hash/8613985ec49eb8f757ae6439e879bb2a-Abstract.html.

[3] Edoardo M Airoldi et al. *Handbook of mixed membership models and their applications*. CRC press, 2015. DOI: 10.1201/b17520.

[4] Valerio Zerbi et al. "Mapping the mouse brain with rs-fMRI: An optimized pipeline for functional network identification". In: *NeuroImage* 123 (2015), pp. 11–21. DOI: 10.1016/j.neuroimage.2015.07.090.

[5] Joanes Grandjean et al. "Common functional networks in the mouse brain revealed by multi-centre resting-state fMRI analysis". In: *NeuroImage* 205 (2020). DOI: 10.1016/j.neuroimage.2019.116278.

[6] Prem Gopalan and David M. Blei. "Efficient discovery of overlapping communities in massive networks". In: *Proceedings of the National Academy of Sciences* 110 (2013), pp. 14534–14539. DOI: 10.1073/pnas.1221839110.

[7] Bernadette CM Van Wijk, Cornelis J Stam, and Andreas Daffertshofer. "Comparing brain networks of different size and connectivity density using graph theory". In: *PloS one* 5.10 (2010), e13701. DOI: 10.1371/journal.pone.0013701.

[8] Xiaoran Yan et al. "Weight thresholding on complex networks". In: *Phys. Rev. E* 98 (4 Oct. 2018), p. 042304. DOI: 10.1103/PhysRevE.98.042304.

[9] Quanxin Wang et al. "The Allen Mouse Brain Common Coordinate Framework: A 3D Reference Atlas". In: *Cell* 181 (2020), 936–953.e20. DOI: 10.1016/j.cell.2020.04.007.

[10] Kristoffer J Albers et al. "Using connectomics for predictive assessment of brain parcellations". In: *NeuroImage* 238 (2021), p. 118170. DOI: 10.1016/j.neuroimage.2021.118170.

[11] Ofer Yizhar et al. "Optogenetics in Neural Systems". In: *Neuron* 71 (2011), pp. 9–34. DOI: 10.1016/j.neuron.2011.06.004.

[12] William E Allen et al. "Global representations of goal-directed behavior in distinct cell types of mouse neocortex". In: *Neuron* 94.4 (2017), pp. 891–907. DOI: 10.1016/j.neuron.2017.04.017.

[13] Chi Ren and Takaki Komiyama. "Characterizing cortex-wide dynamics with wide-field calcium imaging". In: *Journal of Neuroscience* 41.19 (2021), pp. 4160–4168. DOI: 10.1523/JNEUROSCI.3003-20.2021.

[14] Daniel Barson et al. "Simultaneous mesoscopic and two-photon imaging of neuronal activity in cortical circuits". In: *Nature methods* 17 (2019), pp. 107–113. DOI: 10.1038/s41592-019-0625-2.

[15] Andrew J. Peters et al. "Striatal activity topographically reflects cortical activity." In: *Nature* (2021). DOI: 10.1038/s41586-020-03166-8.

[16] Roger Guimerà and Luis A. Nunes Amaral. "Functional cartography of complex metabolic networks". In: *Nature* 433 (2005), pp. 895–900. DOI: 10.1038/nature03288.

[17] Martijn P van den Heuvel and Olaf Sporns. "Network hubs in the human brain". In: *Trends in cognitive sciences* 17.12 (2013), pp. 683–696. DOI: 10.1016/j.tics.2013.09.012.

[18] Jonathan D. Power et al. "Evidence for Hubs in Human Functional Brain Networks". In: *Neuron* 79 (2013), pp. 798–813. DOI: 10.1016/j.neuron.2013.07.035.

[19] Adam Liska et al. “Functional connectivity hubs of the mouse brain”. In: *NeuroImage* 115 (2015), pp. 281–291. DOI: 10.1016/j.neuroimage.2015.04.033.

[20] Maxwell A. Bertolero, B. T. Thomas Yeo, and Mark D’Esposito. “The diverse club”. In: *Nature Communications* 8 (2017). DOI: 10.1038/s41467-017-01189-w.

[21] Jeffrey C Erlich, Max Bialek, and Carlos D Brody. “A cortical substrate for memory-guided orienting in the rat”. In: *Neuron* 72.2 (2011), pp. 330–343. DOI: 10.1016/j.neuron.2011.07.010.

[22] Tsai-Wen Chen et al. “A Map of Anticipatory Activity in Mouse Motor Cortex”. In: *Neuron* 94 (2017), 866–879.e4. DOI: 10.1016/j.neuron.2017.05.005.

[23] Julie A Harris et al. “Hierarchical organization of cortical and thalamic connectivity”. In: *Nature* 575.7781 (2019), pp. 195–202. DOI: 10.1038/s41586-019-1716-z.

[24] Ying Ma et al. “Resting-state hemodynamics are spatiotemporally coupled to synchronized and symmetric neural activity in excitatory neurons”. In: *Proceedings of the National Academy of Sciences* 113 (2016), E8463–E8471. DOI: 10.1073/pnas.1525369113.

[25] Evelyn M. R. Lake et al. “Simultaneous cortex-wide fluorescence Ca^{2+} imaging and whole-brain fMRI”. In: *Nature methods* 17 (2020), pp. 1262–1271. DOI: 10.1038/s41592-020-00984-6.

[26] Andrea Lancichinetti and Santo Fortunato. “Benchmarks for testing community detection algorithms on directed and weighted graphs with overlapping communities”. In: *Physical review. E, Statistical, nonlinear, and soft matter physics* 80 1 Pt 2 (2009), p. 016118. DOI: 10.1103/PhysRevE.80.016118.
