# Supplementary material for: Multimodal measures of spontaneous brain activity reveal both common and divergent patterns of cortical functional organization
Source: Nat Commun. 2024 Jan 3;15:229. doi: 10.1038/s41467-023-44363-z (PMC10764905; doi:10.1038/s41467-023-44363-z)
Supplement: Supplementary file 3 — Reporting Summary [file 41467_2023_44363_MOESM3_ESM.pdf]

nature portfolio

Corresponding author(s): Hadi Vafaii

Last updated by author(s): 2023/11/04

Reporting Summary

Nature Portfolio wishes to improve the reproducibility of the work that we publish. This form provides structure for consistency and transparency in reporting. For further information on Nature Portfolio policies, see our Editorial Policies and the Editorial Policy Checklist.

Statistics

For all statistical analyses, confirm that the following items are present in the figure legend, table legend, main text, or Methods section.

n/a	Confirmed	
☐	☒	The exact sample size (*n*) for each experimental group/condition, given as a discrete number and unit of measurement
☐	☒	A statement on whether measurements were taken from distinct samples or whether the same sample was measured repeatedly
☐	☒	The statistical test(s) used AND whether they are one- or two-sided<br>*Only common tests should be described solely by name; describe more complex techniques in the Methods section.*
☐	☒	A description of all covariates tested
☐	☒	A description of any assumptions or corrections, such as tests of normality and adjustment for multiple comparisons
☐	☒	A full description of the statistical parameters including central tendency (e.g. means) or other basic estimates (e.g. regression coefficient) AND variation (e.g. standard deviation) or associated estimates of uncertainty (e.g. confidence intervals)
☐	☒	For null hypothesis testing, the test statistic (e.g. *F*, *t*, *r*) with confidence intervals, effect sizes, degrees of freedom and *P* value noted<br>*Give P values as exact values whenever suitable.*
☒	☐	For Bayesian analysis, information on the choice of priors and Markov chain Monte Carlo settings
☐	☒	For hierarchical and complex designs, identification of the appropriate level for tests and full reporting of outcomes
☐	☒	Estimates of effect sizes (e.g. Cohen's *d*, Pearson's *r*), indicating how they were calculated

Our web collection on statistics for biologists contains articles on many of the points above.

Software and code

Policy information about availability of computer code

Data collection	The magnetic resonance imaging (MRI) data were collected on a Bruker 11.7T magnet using ParaVision 6.0.1 software. MRI and calcium fluorescence recordings were synchronized using Spike2 7.07. Calcium fluorescence data were recorded using CamWare V3.17. Illumination was controlled by LLE 7Ch Controller from lumencor.
Data analysis	- Code to produce all the results in the paper (https://github.com/hadivafaii/Ca-fMRI; DOI: 10.5281/zenodo.10055413)<br>- Network analysis: SVINET (master branch, v2015) (github.com/premgopalan/svinet; DOI: 10.1073/pnas.1221839110)<br>- Allen SDK v2.12.3, used in our ROI delineation method (https://allensdk.readthedocs.io/en/latest/)<br>- Code by Knox et al., 2018, used in our ROI delineation method (https://github.com/AllenInstitute/mouse_connectivity_models; DOI: 10.1162/netn_a_00066)<br>- LFR synthetic graphs (santofortunato.net/resources, "package 1, undirected and unweighted graphs with overlapping communities"; DOI: 10.1103/PhysRevE.80.016118)<br>- Multimodal data registration: BioImage Suite (bioimagesuite.com)<br>- fMRI preprocessing: RABIES v0.4.2 (github.com/CoBrALab/RABIES; DOI: 10.1101/2022.08.20.504597)<br>- Ca2+ preprocessing: a pipeline previously published by us (github.com/YaleMRRC/calPrep; DOI: 10.1016/j.neuroimage.2022.119735)<br>- Statistical analysis, including permutation tests and bootstrapping: scipy.stats v1.7.0 (docs.scipy.org/doc/scipy/reference/stats.html)-Multiple comparison correction: Python statsmodels v0.13.5 (statsmodels.org/dev/generated/statsmodels.stats.multitest.multipletests.html)<br>- Python scientific computing environments: NumPy v1.20.2, scikit-learn v1.2.0, pandas v1.3.3, matplotlib v3.7.1, seaborn v0.10.1

For manuscripts utilizing custom algorithms or software that are central to the research but not yet described in published literature, software must be made available to editors and reviewers. We strongly encourage code deposition in a community repository (e.g. GitHub). See the Nature Portfolio guidelines for submitting code & software for further information.

Data

Policy information about availability of data

All manuscripts must include a data availability statement. This statement should provide the following information, where applicable:

- Accession codes, unique identifiers, or web links for publicly available datasets
- A description of any restrictions on data availability
- For clinical datasets or third party data, please ensure that the statement adheres to our policy

The raw data analyzed in the present study is available under restricted access because of the complexity of the multimodal data structure and the size of the data. Access can be obtained by contacting the corresponding authors. We will respond to reasonable requests for data access in a timely manner. Data from the Allen Reference Atlas and CCFv3 are available on their website (https://portal.brain-map.org/) or through the Allen Software Development Kit (https://allensdk.readthedocs.io/en/latest/). Source data are provided with this paper.

Research involving human participants, their data, or biological material

Policy information about studies with human participants or human data. See also policy information about sex, gender (identity/presentation), and sexual orientation and race, ethnicity and racism.

Reporting on sex and gender	n/a
Reporting on race, ethnicity, or other socially relevant groupings	n/a
Population characteristics	n/a
Recruitment	n/a
Ethics oversight	n/a

Note that full information on the approval of the study protocol must also be provided in the manuscript.

Field-specific reporting

Please select the one below that is the best fit for your research. If you are not sure, read the appropriate sections before making your selection.

☒ Life sciences ☐ Behavioural & social sciences ☐ Ecological, evolutionary & environmental sciences

For a reference copy of the document with all sections, see nature.com/documents/nr-reporting-summary-flat.pdf

Life sciences study design

All studies must disclose on these points even when the disclosure is negative.

Sample size	The sample size of n = 10 mice was chosen based on the standards of the field. This is in line with what is typical for fMRI studies in rodents (e.g., n = 10 from Daniel Gutierrez-Barragan et al., 2022; DOI: 10.1016/j.cub.2021.12.015) and greater than what is typical for wide-field fluorescence imaging. Since these analyses were conducted on a dataset that was already acquired, we did not have control over the sample size. Additionally, our group results were reproduced at the level of the individuals (Supplementary Fig. 4) lending support for the robustness of our group results with n = 10 animals.
Data exclusions	Data were scrubbed for motion using a conservative 0.1 mm threshold. High-motion frames were selected based on estimates from the fMRI time series and applied to both fMRI and Ca2+ data. Runs were removed from the data pool if half of the imaging frames exceed this threshold for a given run. In this dataset, 2 runs (or around 1.7% of all runs) were removed for this reason. Additionally, 2 more runs were removed because they did not pass our quality control criteria.
Replication	To ensure the robustness of our findings, we repeated our analysis using a large combination of parameters, including the number of networks, regions of interest granularity, graph density, and fMRI preprocessing pipeline. All attempts at replication were successful and our results were qualitatively reproduced across all conditions. See Table 1 in the manuscript for more info and reference to relevant figures.
Randomization	This study aims to characterize large-scale functional networks of the mouse cortex measure during resting-state. Thus, all mice belonged to the same group and no randomization was needed.
Blinding	Blinding was not relevant to this study as all mice were considered in the same experimental group.

Reporting for specific materials, systems and methods

We require information from authors about some types of materials, experimental systems and methods used in many studies. Here, indicate whether each material, system or method listed is relevant to your study. If you are not sure if a list item applies to your research, read the appropriate section before selecting a response.

Materials & experimental systems

n/a	Involved in the study
☒	☐ Antibodies
☒	☐ Eukaryotic cell lines
☒	☐ Palaeontology and archaeology
☐	☒ Animals and other organisms
☒	☐ Clinical data
☒	☐ Dual use research of concern
☒	☐ Plants

Methods

n/a	Involved in the study
☒	☐ ChIP-seq
☒	☐ Flow cytometry
☐	☒ MRI-based neuroimaging

Animals and other research organisms

Policy information about studies involving animals; ARRIVE guidelines recommended for reporting animal research, and Sex and Gender in Research

Laboratory animals	Mice: SLC, Slc17a7-cre/Camk2α-tTA/TITL-GCaMP6f also known as Slc17a7-cre/Camk2αtTA/Ai93. Animals were 6-8 weeks old, 25-30g, at the time of the first imaging session. Mice were generated from parent 1 (Slc17a7-IRES2-Cre-D) and parent 2 (Ai93(TITL-GCaMP6f)-D;CaMK2a-tTA). Both were on a C57BL/6J background. To generate these animals, male CRE mice were selected from the offspring of parents with different genotypes, which is necessary to avoid leaking of CRE expression. Animals were originally purchased from the Jackson Laboratory.
Wild animals	No wild animals were used.
Reporting on sex	Sex information was not collected. Sample was mixed sex.
Field-collected samples	No field collected samples were used.
Ethics oversight	All procedures were approved by the Yale Institutional Animal Care and Use Committee (IACUC) and followed the National Institute of Health Guide for the Care and Use of Laboratory Animals. All surgeries were performed under anesthesia.

Note that full information on the approval of the study protocol must also be provided in the manuscript.

Magnetic resonance imaging

Experimental design

Design type	Resting state
Design specifications	Each mouse was imaged for 3 sessions, with 4 runs per session each lasting 10 minutes (Figure 1b).
Behavioral performance measures	n/a

Acquisition

Imaging type(s)	Functional and structural
Field strength	11.7 T
Sequence & imaging parameters	Functional magnetic resonance imaging (fMRI) data were collected using a gradient-echo, echo-planar-imaging (EPI) sequence with a repetition time (TR) of 1 second, and echo time (TE) of 9ms. EPI data were collected at 0.4x0.4x0.4mm3 resolution, across 28 slices. The 28 slices cover from the middle of the olfactory bulb to the middle of the cerebellum, providing effective whole-brain coverage. Each EPI acquisition was 600 repetitions in length (10 minutes). From each mouse, we collected four resting-state runs. In addition, we collected a high in-plane resolution image of the fMRI field of view (FOV) using a multi-spin-multi-echo (MSME) imaging sequence. In 10 minutes, 40 seconds, using a TR/TE of 2500/20ms, we obtained 28 slices (0.4mm thick) with an in-plane resolution of 0.1x0.1mm2 (two averages). We also collected an isotropic 3D anatomy image of the whole brain using a MSME imaging sequence. In 5 minutes, 20 seconds, using a TR/TE of 5500/15ms, we obtain a 0.2x0.2x0.2mm3 (single average) image of the whole brain (including the entire olfactory bulb and cerebellum which are needed for registration purposes). This sequence was repeated throughout our imaging protocol interleaved with functional acquisitions. Interleaving allows recovery of the calcium signal and more robust responses to stimulation. We also acquire a time-of-flight angiogram using a fast-low-angle-shot (FLASH) imaging sequence. In 18 minutes, using a TR/TE of 130/4ms, we obtain a 0.05x0.05x0.05mm3 2.0x1.0x2.5cm3 image of the blood vessels within the cortex. Finally, we acquire a high resolution anatomy image of the angiogram FOV. Also using a FLASH sequence. In 7 minutes, 30 seconds, using a TR/TE of 61/7.5ms, we obtain an image

	with resolution 0.13x0.08x0.05mm3 and FOV 2.0x1.0x2.5cm3 capturing the anatomy within the same FOV as the MR-angiogram.
Area of acquisition	Depending on the purpose of each scan, we collected data from the whole brain or part of the brain. Please refer to the description of the sequences and imaging parameters provided above.

Diffusion MRI ☐ Used ☒ Not used

Preprocessing

Preprocessing software	RABIES v0.4.2
Normalization	Functional data were not normalized. Instead, regions of interest (ROI) were defined within a common space and the ROIs were transformed back into the individual spaces to perform group analysis.
Normalization template	Allen Mouse Brain Common Coordinate Framework (CCFv3)
Noise and artifact removal	RABIES: github.com/CoBrALab/RABIES; DOI: 10.1101/2022.08.20.504597
Volume censoring	Data were scrubbed for motion using a 0.1 mm threshold.

Statistical modeling & inference

Model type and settings	*Specify type (mass univariate, multivariate, RSA, predictive, etc.) and describe essential details of the model at the first and second levels (e.g. fixed, random or mixed effects; drift or auto-correlation).*
Effect(s) tested	*Define precise effect in terms of the task or stimulus conditions instead of psychological concepts and indicate whether ANOVA or factorial designs were used.*

Specify type of analysis: ☐ Whole brain ☒ ROI-based ☐ Both

Anatomical location(s)	Cortical surface. We aimed to compare network structure across modalities using the same subset of regions. Thus, analyses were restricted to ROIs that appeared in the Ca2+ imaging FOV after multimodal co-registration.
Statistic type for inference (See Eklund et al. 2016)	*Specify voxel-wise or cluster-wise and report all relevant parameters for cluster-wise methods.*
Correction	*Describe the type of correction and how it is obtained for multiple comparisons (e.g. FWE, FDR, permutation or Monte Carlo).*

Models & analysis

n/a	Involved in the study
☐	☒ Functional and/or effective connectivity
☐	☒ Graph analysis
☒	☐ Multivariate modeling or predictive analysis

Functional and/or effective connectivity	Pearson correlation
Graph analysis	Binarized graphs inferred for each run at individual level. Network structure reported both at group and individual levels. Local measures (node centrality measures including degree and entropy) as well as global measures (community structure) reported.
